# Performance Analysis of Two-Way Satellite-Terrestrial Relay Network with SWIPT

**DOI:** 10.3390/s21134303

**Published:** 2021-06-23

**Authors:** Zhen Li, Mingchuan Yang, Gang Wang

**Affiliations:** 1 Communication Research Center, Harbin Institute of Technology, Harbin 150001, China; 16b305001@hit.edu.cn (Z.L.); gwang51@hit.edu.cn (G.W.); 2 Science and Technology on Communication Networks Laboratory, Shijiazhuang 050002, China

**Keywords:** satellite-terrestrial relay network, simultaneous wireless information and power transfer (SWIPT), selective physical-layer network coding (SPNC), Shadowed-Rician fading

## Abstract

In this paper, we investigated the performance of a two-way satellite-terrestrial DF relay network with asymmetric simultaneous wireless information and power transfer (SWIPT). In particular, selective physical-layer network coding (SPNC) was employed in the proposed network, improving the throughput performance. We derived the expressions of system average end-to-end throughput and single node detection (SND) occurrence probability. Furthermore, to observe the effects of the power splitting (PS) coefficient on the energy efficiency performance, the expressions of energy harvested in the physical-layer network coding (PNC) and SPNC protocol were also derived. Finally, theoretical analyses and Monte Carlo simulation results are presented to show: (i) SPNC protocol outperforms the conventional PNC protocol in the two-way satellite-terrestrial relay network with SWIPT in infrequent light shadowing (ILS), average shadowing (AS), and frequent heavy shadowing (FHS) Shadowed-Rician fading channels; (ii) as the channel state gets worse, SPNC protocol can achieve more performance improvement than PNC protocol; (iii) as the PS coefficient increases, the average end-to-end throughput performance increases progressively, and the average energy efficiency performance increases progressively within a certain range, while decreasing in the others.

## 1. Introduction

Land mobile satellite (LMS) communication systems can provide accessibility and high-speed broadcast access for global users, especially in reducing broadband cost, navigation, emergency relief, etc. The hybrid satellite-terrestrial network (HSTN) has received tremendous attention due to its performance advantage and space advantage. In [1], the authors proposed the satellite-terrestrial network. Significant progress on Shadowed-Rician fading channel research has already been made in LMS communication systems. The closed-form expressions of the probability density function (PDF) and the cumulative distribution function (CDF) for the signal-to-noise ratio (SNR) in Shadowed-Rician fading channels were derived in [2]. In order to effectively overcome the severe shadowing effect of satellite-terrestrial links, a hybrid satellite-terrestrial relay network was proposed in [3,4]. Considering unacceptable time delay during the satellite-terrestrial link in a conventional four time-slot communication, two-way relay technology was introduced into the hybrid satellite-terrestrial relay network. In [5], the authors applied analog network coding (ANC) and PNC technology to the bidirectional satellite-terrestrial network. In [6], the concept of PNC is introduced in the bidirectional relay channel, where the two users are communicating with each other through an intermediate node or relay. Both the users transmit their messages to the relay simultaneously. Exclusive OR (XOR) operation is applied at the relay for broadcasting the XORed messages to both users. In [7], the authors proposed a novel PNC protocol, named SPNC, in which SPNC protocol performs better than conventional PNC protocol in throughput performance over Rayleigh fading channel, where the XOR operation is replaced by single node detection during the second time slot of conventional PNC. In [8], the authors derived upper bounds in closed-form for average BERs of the SPNC scheme over Rayleigh fading channels. In [9], the exact BER analysis of PNC for two-way relay channels was presented by the authors. In [10], the effect of hardware impairments on a two-way satellite-terrestrial relay network was analyzed. An opportunistic relay selection scheme was employed in a two-way satellite multi-terrestrial cooperative network in [11]. In [12], the satellite-terrestrial cooperative network was analyzed. The performance of a distributed space-time coding-based hybrid satellite-terrestrial cooperative system with a single fixed terrestrial relay was investigated. However, the lifetime of a two-way satellite-terrestrial network is limited by the battery power of the terrestrial mobile user.

To overcome the problem of battery limitation, we can introduce energy harvesting techniques to extend the system’s lifetime. The energy-constrained device can harvest energy from a portion of the received signal without affecting the communication performance. The author proposed SWIPT in [13], where the receiver simultaneously executes information decoding and energy harvesting. Considering the structure of the wireless receiver, [14] proposed two kinds of effective working modes: time switching (TS) mode and PS mode. PS mode uses one portion of the received signal power for information decoding and the remaining portion for energy harvesting from the perspective of power allocation in [15,16]. From the perspective of time slot allocation, TS mode divides a communication period into two portions in [17,18], one of which is used for information decoding. The remaining portion is used for energy harvesting. Much research work has been done on relay energy harvesting scenarios [19,20,21]. In [22], an asymmetric energy harvesting model was discussed with energy efficiency (EE) precoding design in MIMO two-way relay network. EE was defined as the ratio of total energy consumption and information rate in green communications in [23,24,25]. Especially in a network composed of the energy-constrained device, EE will be an indispensable metric to measure system performance.

However, the existing theoretical analyses on energy harvesting are focused on terrestrial communication systems. To solve the problem, we introduced the SWIPT technology into account and investigate the performance of the SWIPT aided two-way satellite-terrestrial relay network.

Firstly, we propose the framework of the SWIPT aided two-way satellite-terrestrial relay network model and introduce the SPNC protocol to improve the throughput performance of the proposed network. Before deriving exact average end-to-end throughput expressions of PNC and SND protocols, we derive the exact BERs and instantaneous throughputs. Secondly, we derive the probability of single node detection occurrence of SPNC protocol in the SWIPT aided system. Thirdly, to get the energy efficiency performance of the SWIPT aided system, the energy harvesting at the user source node is given.

The rest of this paper is organized as follows: In Section 2, the SWIPT aided two-way satellite-terrestrial relay network model is presented, and a brief introduction to SPNC protocol is given. In Section 3, the system performance of the proposed network is investigated. In Section 4, Monte Carlo simulation results are provided to verify the correctness of theoretical results, and the conclusion of this paper is summarized in Section 5.

## 2. System Model and Selective PNC

**Figure 1:**
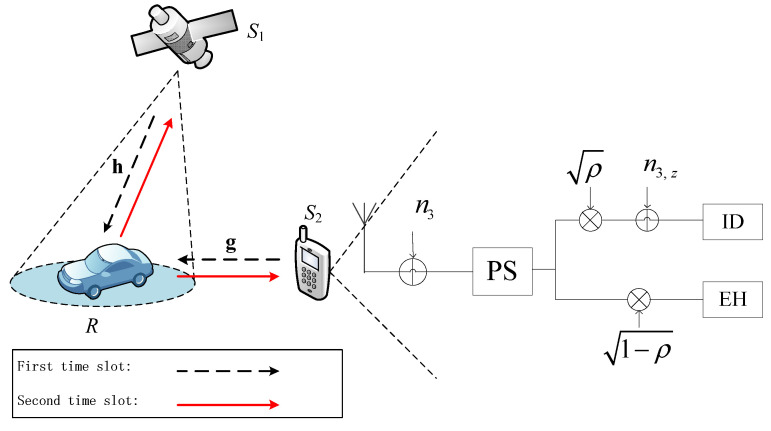
Illustration of the system model.

As shown in Figure 1, we consider a SWIPT enabled two-way DF satellite-terrestrial network, with a satellite source node (*S*_1_) and an energy-constrained user source node (*S*_2_) at ground exchange information with the assistance of a mobile terminal relay node (*R*) situated at the ground. Both the SPNC protocol and PS scheme are employed in the two-way satellite-terrestrial network. We assume the satellite source node, user source node, and mobile terminal relay node have a single antenna and operate in half-duplex mode. Therefore, there is no direct link between the satellite source node and the user source node.

In the proposed network, non-identical, independent, and reciprocal fading channels are assumed. The satellite–mobile terminal link is modeled as Shadowed-Rician fading channel. The mobile terminal can receive the signal from the line of sight (LOS) and the signal from other paths. The destination–mobile terminal link is modeled as Rayleigh fading channel, which is a multipath scene. In Figure 1, *g* and *h* denote fading channel coefficients of links *S*_1_—*R* (or *R*—*S*_1_) and *S*_2_—*R* (or *R*—*S*_2_), respectively. Hence, *g* is the independent and identically distributed Shadowed-Rician random variable (RV), and *h* is the independent and identically distributed Rayleigh RV. Specifically, channel state information (CSI) is only available on the receiver side.

It takes two time slots for the communication. Let *T* be the duration of the entire transmission block, divided into two time slots with a time proportion factor β∈(0,1). During the first time slot of duration βT, the satellite or the user simultaneously transmits the signals *s*_1_ or *s*_2_ to the mobile terminal with the transmit power *P*_1_ or *P*_2_, respectively, and the received signal at the relay is

(1) $$r_{1}=h\sqrt{P_{1}}s_{1}+g\sqrt{P_{2}}s_{2}+n_{1}$$

where the additive noise *n*_1_ indicates the complex Gaussian noise with zero mean and variance $W\sigma^{2}$; *W* denotes the system bandwidth; *h* is the channel coefficient between *S*_1_ and *R*; *g* is the channel coefficient between *R* and *S*_2_. The transmit signals are generated corresponding to the messages *m*_1_ and *m*_2_ from *S*_1_ and *S*_2_, respectively. Assume that the BPSK signaling is employed by both *S*_1_ and *S*_2_. The BPSK mapping follows *s_i_* = 1–2*m_i_* for *i* = 1,2. After receiving, decision making is according to the maximum likelihood (ML) detection rule; the message $\hat{m}$ is equal to 0 if

(2) $$\begin{array}{l} \sum_{\hat{m}=0}\exp\left[\left|r_{1}-h\sqrt{P_{1}}s_{1}(m_{1})-g\sqrt{P_{2}}s_{2}(m_{2})\right|\right]\\ \;\;\;\;\;\;>\sum_{\hat{m}=1}\exp\left[\left|r_{1}-h\sqrt{P_{1}}s_{1}(m_{1})-g\sqrt{P_{2}}s_{2}(m_{2})\right|\right] \end{array}$$

or $\hat{m}=1$ otherwise, where $\hat{m}=m_{1}⊕m_{2}$.

During the second time slot of duration (1−β)T, *R* broadcasts $s_{R}$ to *S*_1_ and *S*_2_ with the transmit power *P_R_*, which follows the function $s_{R}=1–2\hat{m}$. The received signal at *S*_1_ and *S*_2_ can be expressed as $y_{1}=h\sqrt{P_{1}}s_{R}+n_{2}$ and $y_{2}=g\sqrt{P_{1}}s_{R}+n_{3}$, respectively, where the additive noise *n*_2_ and *n*_3_ indicate the complex Gaussian noise with zero mean and variance $W\sigma^{2}$. After receiving signal *y*_2_ from *R*, *S*_2_ splits it into two parts with ratio ρ, where the portion $\sqrt{\rho }y_{2}$ is used for information decoding and the remaining $\sqrt{1-\rho }y_{2}$ for RF energy harvesting. The received signal at *S*_2_ for decoding information and the harvested energy during the second time slot (1−β)T can be respectively written as:

(3) $$y_{2,ID}=\sqrt{\rho }\left(g\sqrt{P_{R}}s_{R}+n_{3}\right)+n_{3,z}$$

(4) $$E_{S_{2},EH}=\eta (1-\beta )T(1-\rho )P_{R}{\left|g\right|}^{2}$$

where η is the energy conversion efficiency, the additive noise $n_{3,z}$ indicates the complex Gaussian noise with zero mean and variance $W\sigma^{2}$.

After employing the minimum Euclidean distance rule, *S*_1_ and *S*_2_ can detect ${\tilde{m}}_{i}$ as ${\tilde{m}}_{2}=\underset{\hat{m}\in \left(0,1\right)}{\arg\min}{\left|y_{1}-h\sqrt{P_{R}}s_{R}\right|}^{2}$ and ${\tilde{m}}_{1}=\underset{\hat{m}\in \left(0,1\right)}{\arg\min}{\left|y_{2,ID}-\sqrt{\rho }g\sqrt{P_{R}}s_{R}\right|}^{2}$, respectively. Then, the satellite source node and the user source node can detect the message by applying XOR operation on ${\tilde{m}}_{i}$ with its own message *m_i_*.

### 2.1. Selective PNC

**Figure 2:**
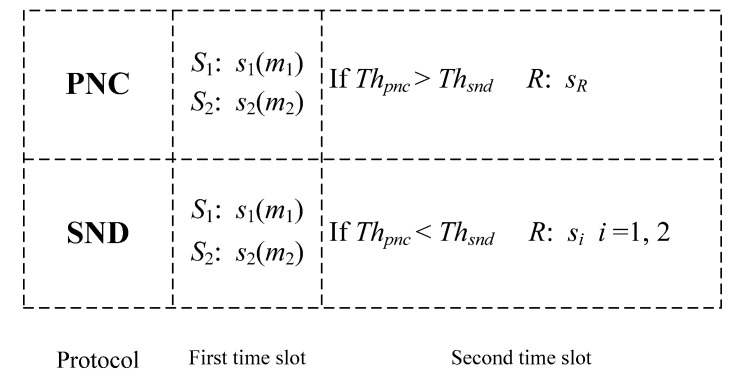
Description of selective PNC protocol. During the first time slot, the satellite source node and the user source node transmit messages *S*_1_ and *S*_2_, respectively. During the second time slot, the mobile terminal relay broadcast and forward *S_R_* in PNC protocol, while broadcast and forward one of *S*_1_ and *S*_2_, according to the channel coefficient in SND protocol.

In the conventional PNC protocol, the mobile terminal simultaneously broadcasts the signal $s_{R}$ to both the satellite source node and the user source node during the second time slot. However, in selective PNC protocol, to outperform conventional PNC protocol in the sense of average end-to-end throughput, we introduce the single node detection protocol. The mobile terminal detects messages from the superimposed signal as conventional PNC protocol or detects the message from the better channel by treating the other message as part of the noise. Hence during the second time slot, the mobile terminal can decide according to the instantaneous throughputs for current channel state information as shown in Figure 2.

By employing the SPNC protocol, during the first time slot, the mobile terminal relay compares the value of instantaneous throughput $Th_{pnc}$ with $Th_{snd}$ after receiving the signals *s*_1_ and *s*_2_. If $Th_{pnc}>Th_{snd}$, the mobile terminal relay will detect $\hat{m}$, and broadcast its corresponding BPSK signal $s_{R}$ to both satellite source node and user source node as PNC protocol. If $Th_{pnc}<Th_{snd}$, the mobile terminal will detect the message from the better channel and broadcast its corresponding BPSK signal $s_{i}$ instead of $s_{R}$ to both the satellite source node and the user source node.

During the second time slot, the satellite source node and user source node receive the broadcast signal from the mobile terminal relay. If the broadcast signal is $s_{R}$, the satellite source node and user source node will detect ${\tilde{m}}_{2}$ and ${\tilde{m}}_{1}$ as PNC protocol, respectively. If the broadcast signal is $s_{1}$, the user source node will split it into two parts with ratio ρ, where the portion $\sqrt{\rho }y_{2}$ is used for information decoding and the remaining $\sqrt{1-\rho }y_{2}$ for RF energy harvesting. The received signal at *S*_2_ for decoding information and the harvested energy during the second time slot (1−β)T can be respectively written as:

(5) $$y_{2,ID}=\sqrt{\rho }\left(g\sqrt{P_{R}}s_{1}+n_{3}\right)+n_{3,z}$$

(6) $$E_{S_{2},EH}=\eta (1-\beta )T(1-\rho )P_{R}{\left|g\right|}^{2}$$

If the broadcast signal is $s_{2}$, the satellite source node will detect ${\tilde{m}}_{2}$ as PNC protocol, and the user source node will split the received signal into two parts with ratio $\rho_{0}≃0$. The received signal at the user source node for RF energy harvesting can be written as:

(7) $$E_{S_{2},EH}≃\eta (1-\beta )TP_{R}{\left|g\right|}^{2}$$

### 2.2. Fading Models

In the proposed network, non-identical and independent fading channels are assumed. The satellite source—mobile terminal link is modeled as a Shadowed-Rician fading channel with the following PDF

(8) $$f_{h}(x)=2x\alpha e^{-\mu x^{2}}{}_{1}F_{1}(m;1;\delta x^{2}),\;x>0,$$

where $\alpha =0.5{\left(2bm/\left(2bm+\Omega \right)\right)}^{m}/b$, μ=(0.5/b), $\delta =0.5\Omega /\left(2b^{2}m+b\Omega \right)$, the parameter Ω is the average power of LOS component, 2*b* is the average power of the multipath component, and 0≤m≤∞ is the Nakagami parameter, for m=0 and m=∞, the envelope of *h* follows the Rayleigh and Rician distribution, respectively; and ${}_{1}F_{1}(a;b;z)$ is the confluent hypergeometric function.

The channel of the mobile terminal—destination user link is assumed to follow the Rayleigh distribution g∼CN(0,Ω), where Ω is the average power.

## 3. System Performance Analysis

In this section, the exact expressions for instantaneous end-to-end throughput, average end-to-end throughput, and the proposed network’s energy efficiency with SWIPT for SPNC protocol are obtained, respectively.

### 3.1. Instantaneous BER for SPNC

Before deriving the exact expression for average end-to-end throughput of SPNC protocol, we have to obtain the exact expressions for instantaneous error probability for PNC and SND protocols in the two-way satellite-terrestrial network for BPSK modulation. This is because the instantaneous BERs are affected by the numerical relationship of the *S*_1_—*R* and *S*_2_—*R* links in two cases.

#### 3.1.1. Case 1

In the first case, we assume the *S*_1_—*R* link channel gain is better than the *S*_2_—*R* link channel gain in a block transmission time. The numerical relationship of the links are as follows:

(9) $$\left|g\sqrt{P_{2}}/h\sqrt{P_{1}}\right|=\left|\omega_{0}e^{j\phi }\right|<1$$

Given channel gains, the instantaneous BER of PNC protocol at the mobile terminal relay during the first time slot is written as:

(10) $$p_{MA}^{pnc}\equiv \frac{1}{2}[P(\hat{m}=1|m_{1}⊕m_{2}=0)+P(\hat{m}=0|m_{1}⊕m_{2}=1)]$$

The instantaneous BER of SND protocol at the mobile terminal relay during the first time slot is written as:

(11) $$p_{MA}^{snd}\equiv \frac{1}{2}[P(\hat{m}=1|m_{1}=0)+P(\hat{m}=0|m_{1}=1)]$$

After adopting Craig’s integral used in [26], we can obtain $p_{MA}^{pnc}$ as follows:

(12) $$\begin{array}{l} p_{MA}^{pnc}=Q(\sqrt{\frac{2P_{2}{\left|g\right|}^{2}}{W\sigma^{2}}})+\frac{1}{2}[I_{craig}^{pnc}-Q(\sqrt{\frac{2{\left(2\sqrt{P_{1}}\left|h\right|\cos\phi +\sqrt{P_{2}}\left|g\right|\right)}^{2}}{W\sigma^{2}}})\\ \;\;\;\;\;\;\;\;\;\;\;\;\;\;+Q(\sqrt{\frac{2{\left(2\sqrt{P_{1}}\left|h\right|\cos\phi -\sqrt{P_{2}}\left|g\right|\right)}^{2}}{W\sigma^{2}}})] \end{array}$$

where $I_{craig}^{pnc}$ is as follows:

(13) $$\begin{array}{ll} I_{craig}^{pnc} & =I_{craig}(\sqrt{P_{1}}\left|h\right|,\sqrt{P_{2}}\left|g\right|,\frac{\pi }{2}-\theta +\phi ,\theta -\frac{\pi }{2})\\ & +\;I_{craig}(2\sqrt{P_{1}}\left|h\right|\cos\phi +\sqrt{P_{2}}\left|g\right|,\sqrt{P_{1}}\left|h\right|+2\sqrt{P_{2}}\left|g\right|\cos\phi ,\pi -\nu ,\phi -\pi +\nu )\\ & -\;I_{craig}(\sqrt{P_{2}}\left|g\right|,\left|2\sqrt{P_{2}}\left|g\right|\cos\phi -\sqrt{P_{1}}\left|h\right|\right|,\frac{\pi }{2}-\theta ,\theta -\frac{\pi }{2}+\phi )\\ & -\;I_{craig}(\left|2\sqrt{P_{1}}\left|h\right|\cos\phi -\sqrt{P_{2}}\left|g\right|\right|,\sqrt{P_{1}}\left|h\right|,\phi +\alpha ,-\alpha ) \end{array}$$

where $I_{craig}(a,b,c,d)$ is written as:

(14) $$I_{craig}(a,b,c,d)=\frac{1}{2\pi }\int_{0}^{c}\exp\left[-\frac{a^{2}}{2\sigma^{2}\sin^{2}\varphi }\right]d\varphi +\frac{1}{2\pi }\int_{0}^{d}\exp\left[-\frac{b^{2}}{2\sigma^{2}\sin^{2}\varphi }\right]d\varphi$$

where angle parameters are defined as: $\theta =\arctan\left(\left(\sqrt{P_{1}}\left|h\right|-\sqrt{P_{2}}\left|g\right|\cos\phi \right)/(\sqrt{P_{2}}\left|g\right|\sin\phi )\right)$, $\omega =\arccos\left[\cos\theta \sqrt{P_{1}{\left|h\right|}^{2}-2\sqrt{P_{1}P_{2}}\left|h\right|\left|g\right|\cos\phi +P_{2}{\left|g\right|}^{2}}/\left(\sqrt{P_{2}}\left|g\right|\right)\right]$, α=(3π/2)−ϕ−θ−2ω and $\nu =\left(\pi /2\right)-\phi +\arctan(\left(\sqrt{P_{2}}\left|g\right|\sin(\theta -\phi )/\cos\theta +2\sqrt{P_{2}}\left|g\right|\sin\phi \right)/\left(\sqrt{P_{1}}\left|h\right|+2\sqrt{P_{2}}\left|g\right|\cos\phi \right))$.

Similarly, $p_{MA}^{snd}$ can be obtained as follows:

(15) $$p_{MA}^{snd}=\frac{1}{2}\left[Q(\sqrt{\frac{2B^{2}}{W\sigma^{2}}})+Q(\sqrt{\frac{2A^{2}}{W\sigma^{2}}})-I_{craig}^{snd})\right]$$

where $A=\sqrt{P_{1}{\left|h\right|}^{2}-2\sqrt{P_{1}P_{2}}\left|h\right|\left|g\right|\cos\phi +P_{2}{\left|g\right|}^{2}}$, $B=A+2\sqrt{P_{2}}\left|g\right|\cos(\pi -\theta -\omega )$ and $I_{craig}^{snd}$ is as follows:

(16) $$\begin{array}{ll} I_{craig}^{snd} & =I_{craig}(B,\sqrt{P_{1}}\left|h\right|,\frac{3\pi }{2}-2\theta -\omega ,\theta -\frac{\pi }{2}-\phi )\\ & +\;I_{craig}(\sqrt{P_{1}}\left|h\right|+2\sqrt{P_{2}}\left|g\right|\cos\phi ,B,\pi -\nu -\phi ,\nu -\theta -\omega )\\ & -\;I_{craig}(A,\left|2\sqrt{P_{2}}\left|g\right|\cos\phi -\sqrt{P_{1}}\left|h\right|\right|,\frac{\pi }{2}-\omega ,\frac{\pi }{2}+\phi -\theta )\\ & -\;I_{craig}(\sqrt{P_{1}}\left|h\right|,A,\alpha ,\omega -\frac{\pi }{2}) \end{array}$$

The instantaneous BER of PNC and SND protocols at the satellite source node and user source node during the second time slot are written as, respectively:

(17) $$p_{BC,S_{1}}^{pnc}=Q(\sqrt{\frac{2P_{R}{\left|h\right|}^{2}}{W\sigma^{2}}}),\;p_{BC,S_{2}}^{pnc}=Q(\sqrt{\frac{2\rho P_{R}{\left|g\right|}^{2}}{W\sigma^{2}}})$$

(18) $$p_{BC}^{snd}=Q(\sqrt{\frac{2\rho P_{R}{\left|g\right|}^{2}}{W\sigma^{2}}})$$

#### 3.1.2. Case 2

In the other case, we assume the *S*_2_—*R* link channel gain is better than the *S*_1_—*R* link channel gain in a block transmission time. The relationship of the links are as follows:

(19) $$\left|h\sqrt{P_{1}}/g\sqrt{P_{2}}\right|=\left|\omega_{0}e^{j\phi }\right|<1$$

Given channel gains, the instantaneous BER of PNC protocol at the mobile terminal relay during the first time slot is the same with case 1. Therefore, the instantaneous BER of SND protocol at the mobile terminal relay during the first time slot is written as:

(20) $$p_{MA}^{snd}\equiv \frac{1}{2}[P(\hat{m}=1|m_{2}=0)+P(\hat{m}=0|m_{2}=1)]$$

After adopting Craig’s integral, we can obtain $p_{MA}^{pnc}$ as follows:

(21) $$\begin{array}{l} p_{MA}^{pnc}=Q(\sqrt{\frac{2P_{1}{\left|h\right|}^{2}}{W\sigma^{2}}})+\frac{1}{2}[I_{craig}^{pnc}-Q(\sqrt{\frac{2{\left(2\sqrt{P_{2}}\left|g\right|\cos\phi +\sqrt{P_{1}}\left|h\right|\right)}^{2}}{W\sigma^{2}}})\\ \;\;\;\;\;\;\;\;\;\;\;\;\;\;\;+Q(\sqrt{\frac{2{\left(2\sqrt{P_{2}}\left|g\right|\cos\phi -\sqrt{P_{1}}\left|h\right|\right)}^{2}}{W\sigma^{2}}})] \end{array}$$

where $I_{craig}^{pnc}$ is as follows:

(22) $$\begin{array}{ll} I_{craig}^{pnc} & =I_{craig}(\sqrt{P_{2}}\left|g\right|,\sqrt{P_{1}}\left|h\right|,\frac{\pi }{2}-\theta +\phi ,\theta -\frac{\pi }{2})\\ & +\;I_{craig}(2\sqrt{P_{2}}\left|g\right|\cos\phi +\sqrt{P_{1}}\left|h\right|,\sqrt{P_{2}}\left|g\right|+2\sqrt{P_{1}}\left|h\right|\cos\phi ,\pi -\nu ,\phi -\pi +\nu )\\ & -\;I_{craig}(\sqrt{P_{1}}\left|h\right|,\left|2\sqrt{P_{1}}\left|h\right|\cos\phi -\sqrt{P_{2}}\left|g\right|\right|,\frac{\pi }{2}-\theta ,\theta -\frac{\pi }{2}+\phi )\\ & -\;I_{craig}(\left|2\sqrt{P_{2}}\left|g\right|\cos\phi -\sqrt{P_{1}}\left|h\right|\right|,\sqrt{P_{2}}\left|g\right|,\phi +\alpha ,-\alpha ) \end{array}$$

where angle parameters are defined as: $\theta =\arctan\left(\left(\sqrt{P_{2}}\left|g\right|-\sqrt{P_{1}}\left|h\right|\cos\phi \right)/(\sqrt{P_{1}}\left|h\right|\sin\phi )\right)$, $\omega =\arccos\left[\cos\theta \sqrt{P_{2}{\left|g\right|}^{2}-2\sqrt{P_{1}P_{2}}\left|h\right|\left|g\right|\cos\phi +P_{1}{\left|h\right|}^{2}}/\left(\sqrt{P_{1}}\left|h\right|\right)\right]$, α=(3π/2)−ϕ−θ−2ω and $\nu =\left(\pi /2\right)-\phi +\arctan(\left(\sqrt{P_{1}}\left|h\right|\sin(\theta -\phi )/\cos\theta +2\sqrt{P_{1}}\left|h\right|\sin\phi \right)/\left(\sqrt{P_{2}}\left|g\right|+2\sqrt{P_{1}}\left|h\right|\cos\phi \right))$.

Similarly, $p_{MA}^{snd}$ can be obtained as follows:

(23) $$p_{MA}^{snd}=\frac{1}{2}\left[Q(\sqrt{\frac{2B^{2}}{W\sigma^{2}}})+Q(\sqrt{\frac{2A^{2}}{W\sigma^{2}}})-I_{craig}^{snd})\right]$$

where $A=\sqrt{P_{1}{\left|h\right|}^{2}-2\sqrt{P_{1}P_{2}}\left|h\right|\left|g\right|\cos\phi +P_{2}{\left|g\right|}^{2}}$, $B=A+2\sqrt{P_{1}}\left|h\right|\cos(\pi -\theta -\omega )$ and $I_{craig}^{snd}$ is as follows:

(24) $$\begin{array}{ll} I_{craig}^{snd} & =I_{craig}(B,\sqrt{P_{2}}\left|g\right|,\frac{3\pi }{2}-2\theta -\omega ,\theta -\frac{\pi }{2}-\phi )\\ & +\;I_{craig}(\sqrt{P_{2}}\left|g\right|+2\sqrt{P_{1}}\left|h\right|\cos\phi ,B,\pi -\nu -\phi ,\nu -\theta -\omega )\\ & -\;I_{craig}(A,\left|2\sqrt{P_{1}}\left|h\right|\cos\phi -\sqrt{P_{2}}\left|g\right|\right|,\frac{\pi }{2}-\omega ,\frac{\pi }{2}+\phi -\theta )\\ & -\;I_{craig}(\sqrt{P_{2}}\left|g\right|,A,\alpha ,\omega -\frac{\pi }{2}) \end{array}$$

The instantaneous BER of SND protocol at satellite source node during the second time slot is written as, respectively:

(25) $$p_{BC}^{snd}=Q(\sqrt{\frac{2P_{R}{\left|h\right|}^{2}}{W\sigma^{2}}})$$

### 3.2. Average End-to-End Throughput of SPNC

After obtaining the exact expressions for instantaneous error probability for PNC and SND protocols in the two-way satellite-terrestrial network for BPSK modulation, we can derive the instantaneous throughput of the SPNC protocol according to the throughput definition used in [27], the instantaneous end-to-end throughput of SPNC is given by

(26) $$Th_{spnc}=\max\left(Th_{pnc},Th_{snd}\right)$$

(27) $$Th_{pnc}≃\frac{1}{2}{\left(1-p_{MA}^{pnc}\right)}^{N}{\left(1-p_{BC}^{pnc}\right)}^{N}$$

(28) $$Th_{snd}≃\frac{1}{2}{\left(1-p_{MA}^{snd}\right)}^{N}{\left(1-P_{BC}^{snd}\right)}^{N}$$

where $Th_{pnc}$ is the instantaneous of PNC protocol, $Th_{snd}$ is the instantaneous of SND protocol. *N* is the number of bits long in each packet. Substituting Equations (12), (15), (17), and (18) into Equation (26), we can derive the instantaneous throughput of the SPNC protocol in case 1. Substituting Equations (17), (21), (23), and (25) into Equation (26), we can derive the instantaneous throughput of the SPNC protocol in case 2.

Then the average end-to-end throughput of SPNC is written as:

(29) $$E\left[Th_{spnc}\right]=\int_{z^{pnc}}Th_{pnc}f(h,g)dhdg+\int_{z^{snd}}Th_{snd}f(h,g)dhdg$$

where f(h,g) is joint PDF of *S*_1_—*R* and *S*_2_—*R* link gain. $z^{snd}$ is the *z*-plane region for BPSK defined in [8] written as $z^{snd}=\left\{z|\min(\sqrt{P_{2}}\left|g\right|,\sqrt{P_{1}}\left|h\right|)<\sqrt{W\sigma^{2}\ln(N/6)},\left|ℛ[z]\right|<0.5\right\}$. $z^{pnc}$ is the complementary portion of $z^{snd}$ in *z*-plane. ℛ[⋅] denotes for real part operation.

For the *S*_1_—*R* and *S*_2_—*R* link are independent; we can rewrite Equation (29) as:

(30) $$E\left[Th_{spnc}\right]=\int Th_{pnc}f(g)f(h)dhdg+\int_{z^{snd}}\left(Th_{snd}-Th_{pnc})f(g)f(h\right)dhdg$$

where f(h) is the PDF of *S*_1_—*R* link, which follows Shadowed-Rician fading, f(g) is the PDF of *S*_2_—*R* link, which follows Rayleigh fading.

Substituting Equation (8) into (30), we can obtain the exact expressions of average end-to-end throughput for SPNC protocol. Furthermore, with the help of standard mathematical packets such as Mathematica, which is for numerical and symbolic operations, we can achieve the numerical computation of the confluent hypergeometric function ${}_{1}F_{1}(a;b;z)$. Then we can obtain the numerical results of average end-to-end throughput for SPNC protocol.

### 3.3. Probability of SND in SPNC Protocol

We need to analyze the probability of SND for the system. The probability of SND in SPNC protocol is written as:

(31) $$P_{snd}=\int_{z^{snd}}f(h,g)dhdg$$

Substituting Equation (8) into (31), we can obtain the exact expressions of average end-to-end throughput for SPNC protocol. Furthermore, we can obtain the numerical results with the help of Mathematica.

### 3.4. Energy Efficiency of SPNC Protocol

In this section, we investigate the energy efficiency of the two-way satellite-terrestrial network. Consider the remaining $\sqrt{1-\rho }y_{2}$ is used for RF energy harvesting during the second time slot in PNC and SPNC protocols. We assume user source node can harvest power with additional energy storage equipment. The harvested power $E_{S_{2},EH}$ is written as:

(32) $$E_{S_{2},EH}=\left\{ \begin{array}{l} \eta (1-\beta )T(1-\rho )P_{R}{\left|g\right|}^{2},\;\;\;\mathrm{PNC}\;\mathrm{protocol}\;\&\;\mathrm{case}\;1\;\mathrm{in}\;\mathrm{SND}\;\mathrm{protocol}\;\\ \eta (1-\beta )TP_{R}{\left|g\right|}^{2},\;\;\;\;\;\;\;\;\;\;\;\;\;\;\;\mathrm{case}\;2\;\mathrm{in}\;\mathrm{SND}\;\mathrm{protocol}\; \end{array}\right.$$

The system energy consumption is defined as:

(33) $$E_{sum}=(P_{1}P_{2})\beta T+P_{R}(1-\beta )T-E_{S_{2},EH}$$

According to the energy efficiency $\Theta_{EE}$ definition in [28], we can express the energy efficiency for a two-way satellite-terrestrial network as follows:

(34) $$\Theta_{EE}=\frac{E\left[Th_{spnc}\right]W}{\left(P_{1}+P_{2}\right)\beta T+P_{R}\left(1-\beta \right)T-E_{S_{2},EH}}$$

Substituting Equation (30) into (34), we can obtain the exact expressions of energy efficiency for the SPNC protocol. Moreover, we can obtain the numerical results with the help of Mathematica.

## 4. Numerical Results

In this section, numerical simulation results are provided to verify the theoretical analysis and show the impacts of key parameters on the system performance of the two-way satellite-terrestrial relay network. The satellite source node-mobile terminal relay channel coefficients follow Shadowed-Rician fading distribution. The parameters are listed in Table 1 and follow FHS, AS, and ILS, respectively. The user source node-mobile terminal relay channel coefficients follow Rayleigh fading distribution. During a block transmission time, one data packet is transmitted with *N* = 512 symbols. The PS coefficient *ρ* is 0.5, which means $\sqrt{0.5}$ portion of the received signal in the user source node is used for information decoding, and the remaining $\sqrt{0.5}$ portion of the received signal is harvested. The energy conversion rate η is 0.8.

**Figure 3:**
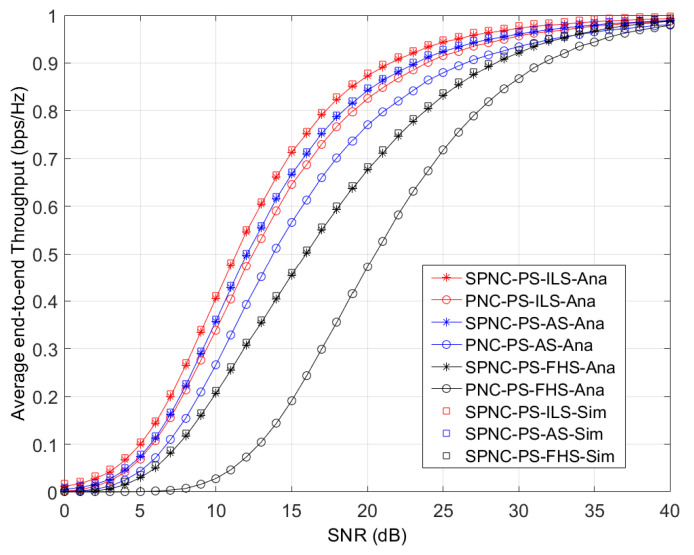
Average end-to-end throughput performance versus the average SNR performance of SPNC and PNC protocols in PS aided two-way satellite-terrestrial relay network with ILS, AS, and FHS.

Figure 3 shows the average end-to-end throughput performance versus the average SNR, which is already normalized with power and distance parameters. In Figure 3, the analytical average end-to-end throughput numerical results match the simulation results. Thus, the throughput performance of the SPNC protocol outperforms one of the PNC protocols in the ILS, AS, and FHS cases, respectively.

**Figure 4:**
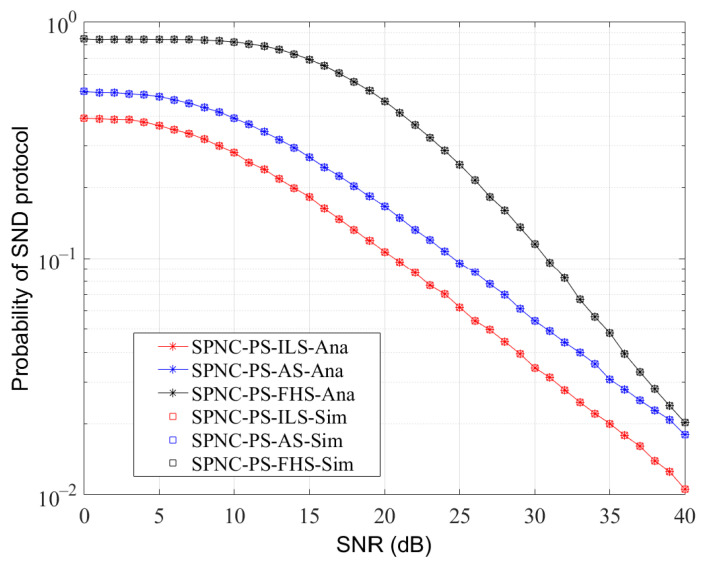
Probability of SND protocol versus the average SNR performance of SPNC protocol in PS aided two-way satellite-terrestrial relay network with ILS, AS, and FHS.

Figure 4 shows the probability of SND protocol versus the average SNR, which is already normalized with power and distance parameters. In Figure 4, the probability of SND protocol numerical results match the simulation results. The probability of SND protocol in the ILS case is significantly higher than the one in AS and FHS cases. This is due to the sharp deterioration of the channel situation. The probability of SND protocol significantly reduces as the gradually increasing of average SNR.

**Figure 5:**
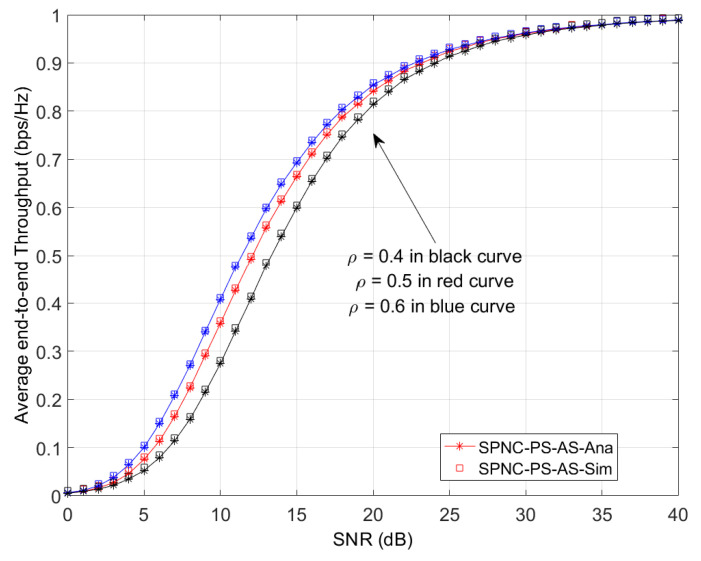
Average end-to-end throughput performance in AS case versus the average SNR with different PS coefficients *ρ*.

Figure 5 shows the average end-to-end throughput performance in AS case versus the average SNR with different PS coefficients *ρ*, which is already normalized with power and distance parameters. In Figure 5, the probability of SND protocol numerical results match the simulation results. The average end-to-end throughput performance in the AS case significantly increases as the PS coefficients *ρ* increase from 0.4 to 0.5 and 0.6. This is due to that the user source node spends more receiving signal energy on informance detection, which increases the SNR of user source node during the second time slot.

**Figure 6:**
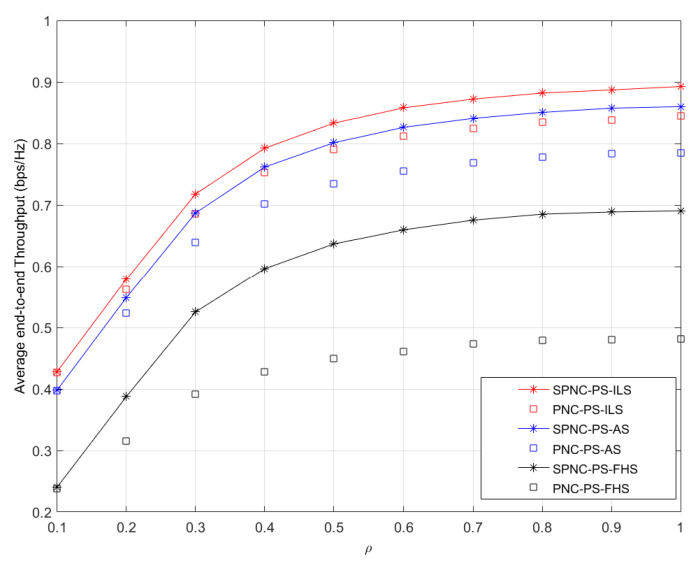
Average end-to-end throughput performance versus different PS coefficients *ρ* in PS aided two-way satellite-terrestrial relay network with ILS, AS, and FHS.

**Figure 7:**
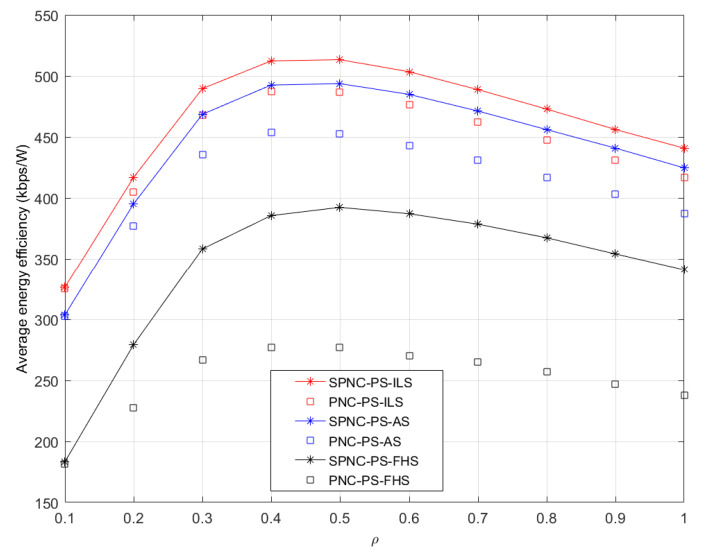
Average energy efficiency performance versus different PS coefficients *ρ* in PS aided two-way satellite-terrestrial relay network with ILS, AS, and FHS.

In Figure 6 and Figure 7, we assume *P*_1_ = *P_R_* = 43 dBm, *P*_2_ = 27 dB. The distance from the satellite source to the mobile terminal is 300 km, and the distance from the user source to the mobile terminal is randomly varying and no more than 50 m. The energy conversion rate η is 0.8. Figure 6 shows the average end-to-end throughput performance versus different PS coefficients *ρ*. The throughput performance of the SPNC protocol outperforms one of the PNC protocols in ILS, AS, and FHS cases, respectively. The average end-to-end throughput performance in three cases significantly increases as the PS coefficient increases. This is because the user source node spends more receiving signal energy on informance detection, which increases the SNR of the user source node during the second time slot.

Figure 7 shows the average energy efficiency performance in AS case versus different PS coefficients *ρ*. The average energy efficiency performance in the ILS case is significantly higher than the one in AS and FHS cases. The average energy efficiency performance in three cases significantly increases as the PS coefficients *ρ* increase from 0.1 to 0.4 or 0.5 and gradually decreases as the PS coefficients *ρ* increase from 0.5 to 1. The increase of average energy efficiency performance from 0.1 to 0.4 or 0.5 is because increasing average end-to-end throughput exceeds the benefit of reducing energy consumption by energy harvesting at the user source node. The decrease of average energy efficiency performance from 0.5 to 1 is due to the benefit from reducing the total energy consumption by energy harvesting at the user source node, which exceeds the benefit from increasing average end-to-end throughput.

## 5. Conclusions

In this paper, we have investigated the performance of the two-way satellite-terrestrial relay network with SWIPT, where both SPNC and PNC protocols have been considered in the system. Firstly, we have derived exact average end-to-end throughput expressions of PNC and SND protocols in the SWIPT aided system. Secondly, we have derived the probability of single node detection occurrence of SPNC protocol in the SWIPT aided system. Thirdly, to get the energy efficiency performance of the SWIPT aided system, the energy harvesting at the user source node has been given. The analysis and simulation results show that: (i) the proposed SPNC protocol outperforms the conventional PNC protocol in the two-way satellite-terrestrial relay network with SWIPT in ILS, AS, and FHS Shadowed-Rician fading channels; (ii) as the channel state gets worse, SPNC protocol can achieve more performance improvement than PNC protocol; (iii) as the PS coefficient increases, the average end-to-end throughput performance increases progressively, and the average energy efficiency performance increases progressively within a certain range while decreasing in the others. The results suggest that if we want to have better comprehensive performance in the SWIPT aided system, we should trade-off between the average end-to-end throughput and the average energy efficiency.

## Figures and Tables

**Table 1:** Land Mobile Satellite channel parameters.

Shadowing	*b*	*m*	Ω
Frequent heavy shadowing (FHS)	0.063	0.739	0.000897
Average shadowing (AS)	0.126	10.1	0.835
Infrequent light shadowing (ILS)	0.158	19.4	1.29

## Data Availability

Not applicable.

## Abbreviations

The following abbreviations are used in this manuscript:

DF	decode-and-forward
SWIPT	simultaneous wireless information and power transfer
SPNC	selective physical-layer network coding
SND	single node detection
PS	power splitting
PNC	physical-layer network coding
ILS	infrequent light shadowing
AS	average shadowing
FHS	frequent heavy shadowing
LMS	land mobile satellite
HSTN	hybrid satellite-terrestrial network
PDF	probability density function
CDF	cumulative distribution function
SNR	signal-to-noise ratio
ANC	analog network coding
XOR	exclusive OR
TS	time switching
EE	energy efficiency
LOS	line of sight
RV	random variable
CSI	channel state information
ML	maximum-likelihood
